# Selections and Abstracts

**Published:** 1906-09

**Authors:** 


### SELECTIONS AND ABSTRACTS.

### THE MECHANICAL TREATMENT OF CONSTIPATION.

By FRED H. MORSE, M.D., Boston, Mass.

Constipation occurs under so many conditions that the successful treatment can be brought about only by just appreciation of the etiology of individual cases. Therefore, I will conform myself to certain conditions in which drugs are worse than useless, and where the proper dietary arrangement avails but little. It is an established fact that among the chief causes of constipation, are those of central nervous origin; including both cerebral and spinal.

Spasmodic conditions in the periphery of the splanchnics, pneumogastric, cervical, or dorsal sympathetic, are sufficient causes to account for constipation. Lack of nerve force, either motor or sensory, whether due to impairment or destructions of histological elements and hysteria, implying a diminished nerve control, can readily explain another cause. Weakness of the muscles of the intestinal and abdominal walls is also a very common factor in the causation of constipation, and becomes a strong therapeutical indication for the modalities, of which I shall speak. With due respect to the dietary and medicinal aspect of our subject, I think that we have in electricity, in its various forms, mechanical vibration and mechanical massage, methods whereby we can accomplish the desired result with a greater degree of certainty, and with more enduring effects than by other procedures understood at present.

It is known that muscles possess an independent power of excitability which may induce more energetic contractions as the result of external stimulation. This may be induced by mechanical stimulation, heat and cold, electricity, or chemical agents. Certain parts, when stimulated, excite muscular contractions; other parts tend to arrest or inhibit movement, and their combined action, when normal, results in a healthy physiological activity. Muscular spasms, then, may result as readily from defective inhibitory action as from an excessive excitability of motor nerve centers, and in the treatment of various spasmodic symptoms for internal medication, this principle is recognized.

To lessen the irritability of the motor centers the bromides are given; to stimulate the inhibitory centers alcohol is administered. It is on this principle, we believe, that combined electrization acts.

The action of electricity for the relief of constipation is twofold: (1) the effect that may be produced by improving the nutrition along the course of the nerves controlling certain parts of the viscera, and (2) its action as a direct stimulus; or in other words, the effect may be produced such as we would desire and expect electricity to have on partially paralyzed muscles in other parts of the body.

By understanding and appreciating the value of the continuous current in its influence upon the circulation, the intelligent use of it in many of the neurotic cases will indirectly cure constipation by its action. The quantity and direction of current varies in the different procedures as in all electrical treatments according to the indications.

Of each subject the technique in every individual case must be carried out with just appreciation of the cause or causes behind it; therefore it is possible, only in a general way, to give the particulars of treatment. But briefly speaking, in neurasthenic cases, where the downward continuous current through the pneumogastric, with a current from two to five ma. employing the interruption in the continuous current (galvanic) from spine to abdomen, using large electrodes, almost always placing the positive one on the back.

The sinusoidal current, and the induced current of high tension, produces a much deeper effect than the usual form of faradic apparatus, or that of low tension.

The slowly interrupted current, as is generally used from the primary coil, producces very little effect as far as electricity is concerned, but can be made useful by virtue of its mechanical action in cases of thin abdominal walls, or where the more powerful mechanical vibration and massage might for various reasons be contraindicated.

The interrupted continuous current with the interrupter adjusted in its undulations to meet the indications of the case in hand, is probably the most potent form of electricity to use in chronic constipation. This may be used with a large abdominal electrode, and the other on the proper part of the spine, or within the rectum, as the individual case demands, taking due care that the seance is not too severe or prolonged to cause unpleasant reaction.

Massage, properly applied, is of great value in treating constipation. In my own work, I prefer the use of a tissue oscillator, having one that can be adjusted to heavy or light, superficial or deep, rapid or slow manipulations to almost any part of the body. It is run by a one-half horsepower motor and consists of a powerful iron frame, to which the patient can be so harnessed as to produce a great variety of motions. This arrangement has proved very efficacious in the treatment of chronic constipation, which was unaccompanied by any conditions of an inflammatory nature, independent of any electrical application. This has been especially observed while treating cases of obesity, where the constipation was only of secondary consideration, and I recall instances where the usual daily cathartic, which had been taken by the patient for years, had been dispensed with.

Vibratory treatment of constipation consists in the manipulation of certain spinal nerve centers, which in many cases can be made very effectual, but rigid attention to the details of this particular modality is all-important, as the usual haphazard indifferent use of many of the vibrators is not only valueless, but may prove harmful. Some of the contraindications for massage and the interrupted continuous current would be the presence of cancer, ulcer, or any acute inflammatory condition.

The application should be made between the transverse processes of the spine, and I think it is usually considered by experts on this subject that the opposite sides treated alternately produce a better effect than if one side were treated completely, then the other.

Physiology teaches us that from the sixth to the twelfth dorsal, stimulation controls the inhibitory action of the small intestine, and of the second, third, fourth, and fifth lumbar and the first, second, and third sacral, induces inhibition of the large intestine, also second, third, fourth, and fifth lumbar, inhibit for descending colon and rectum. The inhibitory nerve of the small intestine is the splanchnic, while the capillaries contain arterial blood; when this changes to venous the splanchnics are stimulated and persistalsis is increased.

In vibrating the abdomen it is essential that the bladder be emptied, the abdominal muscles relaxed by having the patient on his back with knees flexed, and the pressure to be exerted should never be allowed to cause pain. Gradually increasing the rapidity of the stroke, or length of vibrations will usually establish an easy tolerance to the application, compared with giving the full force at once.

The frequency of the séances should be, generally speaking, every day, or every other day for awhile; and gradually increasing the time between treatments, as the results are obtained. In each case, if one has at his command the different kinds of apparatus, which I have mentioned, he will probably find indications for the use of both electrical and mechanical means on the same case, either in conjunction or alternately.

There are three reasons, broadly speaking, why so many are disappointed in the results obtained from the use of physical methods of treatment, and especially electricity, in those cases, where according to well established principles, the treatment ought to be efficacious; (1) ignorance of the physics and physiology of the methods employed; (2) imperfect technique; (3) failure to appreciate the differential indications for the use of the various modalities.

One may possess a thorough knowledge of physics, but if his technique is faulty, this knowledge will avail nothing.—*Journal of Advanced Therapeutics.*

### RELATION OF SYPHILIS WITH JAPANESE RACIAL PECULIARITIES AND CUSTOMS.

By ALBERT S. ASHMEAD, M.D., New York City.

The sensual life is in Japan much more a power of nature than it is in the West.

It is a factor which must be given due consideration if we are to gauge the capacity for development in the Japanese race.

What a text for speculation if we tried to imagine what the progress of the whole East might have been without that tyrannical instinct which enthralls the whole man. Is this to be considered in the light of a reproach to those races? Are our milder instincts meritorious? They themselves think not in spite of the fact that senility comes early to the Japanese.

There are a host of troubles besides a too early senility, which are concomitants and consequences of a most unreasonable sensuality which awakens about the eighteenth or nineteenth year. As soon as the phenomena of puberty become intensified, the Japanese seem to be under the influence of a strange demon; they think hardly of anything but the satisfaction of the new instinct, and waste their time in torpid musings and prurient sensual disquietude. This is the case with females as well as males.

All the love-making of the flower-feasts along the Sumada river, for instance, have for interest the consummation of the sexual passion.

Japanese pederasty is supposed to exist only in the southernmost provinces, in some places which had in former times active intercourse with China. But even this perverted satisfaction of the sexual appetite belongs to the whole of Japan, especially those provinces which have the reputation of producing the most "heroes" are tightly chained to the practice of this vice. A hero, the proverb says, never produces children, because as soon as he has done what secures for him posthumous glory, or certainty of worship as an ancestor, he procures "*bichonen*" (boys) for his sexual gratification instead of using girls, or wife or concubines.

He thus shows that his "heart is strong" enough to resist the blandishments of the inferior *"yo"* (female) principle of *"Kami"* (God) sex. He is "above" normal sexual intercourse which belongs to humans. The Japanese in practicing pederasty want to do something different from the ordinary, common man. The practice of the brothels has more interest for the young man of "New Japan" than has all the wisdom of old "obsolete Christian Europe."

That a man can live virtuously alone without sexual intercourse is a horror to the Japanese mind, and any "Western barbarian" who attempts to do such an awful beastly thing is looked down upon as a monster.

The procurer of girls (a legitimate vocation) will visit you as soon as you step your foot on Japanese soil and will offer to bring you a girl for trial for a month at ten or twenty dollars a month. If you do not like her she will be exchanged at the expiration of the term of trial without extra expense. This is a business just as legitimate in Japan as is that of the "go-between" for marriage. The mikado himself keeps exchanging his twelve concubines.

Every ethnological student of Japan admits that in no people on earth is less store to be laid by its own utterance of its sexual life. All the informations which are brought to us by foreign missionaries and spread abroad by press agents or by the Japanese priests, statesmen and scholars are worthy of double revision and criticism. Even the stories that are told us by numerous globe-trotting travelers, paid press agents, superficially informed doctors, who do not read the Japanese by their own language, and who allow themselves to be gulled, cajoled with their own "superiority" in almost everything, or that are retailed to us by misinformed "press doctors," or those in the pay of the Japanese-London news propaganda and secret service contingent of English politics, who want to read their startling, sensational, hurriedly written effusions before some big American medical meeting, who are so anxious to draw magnetic press attention to their perverted perception of self, or distorted ego—one and all of these stories, as told by these sudden authorities on "things Japanese," are lies cut out of whole cloth.

No people on earth brags about itself as much as the Japanese do, partly through want of philosophical culture or because the majority have thought for so long a time that they are really superior to the Chinese (which they are not in a single thing) or partly from wariness not to expose themselves to censure, but mostly through ingrained national vanity in order that they may not disparage their country in the eyes of us foreigners, who might look down upon them scornfully before they have attained their object of acquiring from us what is necessary to their own idea of political aggrandizement. For example, all the medical gush about their perfect aseptic surgery during the war with Russia, and those hot-water wagons, so recently lauded as original with them, is nothing but what has existed in Japan, imitated from China, since the tea-shrub was first introduced by the Mongolian Buddhism. The Japanese surgeons never, when talking by themselves, I warrant it, give us foreigners the least credit for their haphazard surgical asepsis. There was no induction in its application to wounds by the Japanese imitators. The use of that hot water to the wounded was brought to Manchuria for an entirely different purpose than for wounds. Its virtue in saving their wounded, they claim, was Buddhas', who took a special care of them. They believe that the gods thus protected their god-emperor—the water.

Those wagons with ordinary bathtubs and copper box stoves on wheels supplied to the soldiers, were merely intended to furnish the soldiers with the natural beverage—tea, and to give them their accustomed daily and beloved bath or two.

Buddhist priests of the sixth century had first taught the filth-infected rice-field nation that there was danger from typhoid and distomata in drinking cold (uncooked) water. To the Buddhists, then, and not to Western medicine they claim the credit is due for the good results of the use of aseptic water on the wounds of soldiers made by Christian Russians.

The same argument applies to their use of the hot bath before a battle. They hypocritically claim that it was induction that made them wash before entering a battle. It was not so. Every coolie wanted his one or two hot baths a day, and certainly the soldiers would.

They could not live without hot baths. Most of those hot water wagons were merely intended as perambulating bathing establishments. There was no idea of using them so that they might protect the soldiers going into battle. It was haphazard surgery.

When, therefore, Doctor Suzuko, the chief surgeon of something or other, claims that the Japanese troops were required by their superior surgeons to take a hot bath before entering battle, so that their wounds would not be liable to be poisoned by the dirt of the skin, he exaggerates and states more than should be credited to the Japanese surgical staffs' intentions. The Japanese soldiers on going or not going into a battle would have used the daily hot bath. Even the coolie burden-bearer would insist on taking his hot bath, and it was this only that was the real intention of that portable wagon bath.

Pretense, hypocrisy, prevarication, statistical lying, are all virtues belonging to Japanese officials, in high as well as in low places. The higher the position occupied by a Japanese the greater is the lying. They always assume to be better and wiser than they are.

Cold water is never used by any one in Japan. For a thousand years it has been merely the "warning for the old." It is not to be drunk under any circumstances. "Only old people use it," the proverb says. To drink Japanese cold water, therefore, is to be near death, for it is filled with filth from the rice fields manured with city night stuff. No water that is not boiled was considered for hundreds of years fit to drink *or bathe in.* The wounds of all Japanese (just as do those of Chinese and Turks) heal very quickly and naturally without much inflammation, because of the daily hot baths.

Syphilis in Japan has been influenced in its propagation and course by various peculiarities of race and customs.

The long acquaintance of the people with this disease—since the eleventh century before Christ—in their Chinese ancestry, and since the seventh century of the Christian era more generally over Japan, and the popular belief that syphilization was beneficial, have given the present generations a singular immunity to it. So noticeable is this fact that an European inoculated with the germ has a most virulent type of syphilis, showing that in the Japanese race a peculiar resistance has been built up against the more pronounced symptoms.

The wide-spread practice of concubinage by the middle and upper classes has propagated the disease faster than it otherwise would have been.

The practice of freest prostitution by the lower classes who were unable financially to keep concubines, and the popular belief that marriage with prostitutes brought honour to family strains since the twelfth century, when all the noble women of the Taira family were thrown into prostitution after capture by a warring faction, two of the five great families having fought for possession of the throne for their separate candidate for mikadoship, were reasons for the widespread distribution of syphilis. In those days there were no new-fangled barons, counts and other British imitated titles of nobility. Daimios were the provincial feudal nobles. There was perpetual fighting among themselves for influence at the court of Kioto. They were of more or less white Indonesian descent. The Minamotos had fought the Tairas for four hundred years.

Sometimes there would be two puppet mikados on the throne at a time. Mere boys occupied the throne. The descent of the mikado would be thus perpetuated. Ofttimes a mikado, fearful of his life, or being tired of the strife, would abdicate. Finally, to thwart the Taira cliques, all the women of that family were captured and made prostitutes of. Marrying prostitutes, therefore, gradually became a social custom, for thus the common man thought he might obtain a noble stream of blood in his family's veins. That such women were inoculated with syphilis made no difference. Hence it became the popular belief that syphilis of a wife proved her nobility. It was rather a distinction to be syphilitic, and thus syphilization became a popular practice.

The notorious debaucheries of the Shinto and Buddhist priests acted in the spreading of syphilis. These profligate members of society were supposed to be celibates, but, quite to the contrary, they were practicers of concubinage.

I know that as a class they are grossly immoral, for they formed a very large clientele of my venereal clinic in Tokyo.

The effect resulting from the special racial diet of Japanese of fish and iodized sea-weed helped in building up a national resistance to syphilis.

This diet was a phosphorized diet and made bone highly antagonistic to the tertiary stage of the disorder.

Syphilis appears very mild in its primary and secondary periods. There is not as much tertiary syphilis in Japan as there is in a given number of syphilitics of European blood. Occasionally one meets very severe tertiary syphilis of most virulent type; parietal bones will even slough, and loss of nose septum is extremely common, some of this last in all probability being due to mercurialization, according to the Corean system of inhalation of mercuric fumes and the application of mercuric chloride stuffed in the nostrils. Surely the race has acquired in these days a distinct resistance to the destructive influence of the disease by general syphilization for, say, thirteen hundred years, and by the diet of phosphorized fish and iodized sea-weed.

The widespread debaucheries of the drunken class of Samourai or fighting servant class belonging to every Daimio, their drunken orgies had a great deal to do with the general infusion in the race of the germs of syphilis. These carriers of the two swords, one the long sword to hack at their master's enemy of the next province, and the little sword to commit *hara-kiri* in case of failure in some murderous deed were eternally on the warpath. A Daimio usually had one of these Samourai to carry his sword on a cushion for him. But the servant-men wore theirs strapped about the waist. This was the method of protection or assault of a gentleman's servants. Those lower down in the social scale, beneath a gentleman's or Daimio's servant, were not permitted to wear a sword. This was a privilege pertaining only to a Daimio's or rich man's servant. The lowest classes practiced jiu-jitsu for self-protection.

Only inferior persons ever made use of jiu-jitsu. No gentleman in Japan ever practiced jiu-jitsu; it belonged as a game of war to the lowest blackguard. It was looked upon as the bruiser's method of warfare, beneath even the notice of the servant classes.

We have heard in late times a great deal of the virtues of the *Samourai* or *Bushi*—both words mean the same thing—fighters or soldiers, and *Bushido* or the way or method of the fighting men of Japan has been nauseatingly extolled by the braggarts of the press as a civilized spirit, impelling Japan to noble deeds, but a bigger set of blackguards never existed in the whole world than *Samourai*. Laws were even passed not long before theMeiji era (1867) to protect pregnant women in Japan against assaults by rape of Samourai. Even the belt of women, the "obi," was ordered tied in a distinctive knot in order that respectable women might be protected from assault by Samourai. In fact, the licensing of prostitution was a necessary measure because of those male prostitute's sexual needs.

In the empress' edicts creating schools for young girls it was expressly stipulated that these schools were necessary to protect the growing girls from the debaucheries of the Samourai.

The general use of the Japanese hot bath has had its influence on the propagation of syphilis. The water was rarely changed in the public street baths, where hundreds of syphilitics would bathe and merely protect their sores from view by wearing their *fundoshi* or breech clout. The commonest coolie would take his one or two hot baths daily. The contaminated water would sweat out the mercury, perhaps, which all Mongolians know enough to take as a cure for the disease, for most of the Japanese physicians would follow the old Chinese teachings as to syphilis, especially mercurialization by vapors.

The absence of kissing, as a method of showing loving affection, has served the nation well against propagation of this disease by mucous patches in the syphilitic mouth. However, in the sexual act women will roll their tongues beneath that of the male, and sweep it during coitus around the mouth of the male partner to the act.

The *fundoshi* breech clout, worn by all males, as well as the paper handkerchiefs used by both sexes, the clean in one kimono sleeve and soiled in the other, are a common means of propagation of syphilis. Besides, these paper handkerchiefs are used by menstruating women as a stuffing in the vagina, and when soiled by blood or other discharge are not carefully destroyed as they should be, but are a common means of contamination by being thrown promiscuously anywhere.

The public woman's and man's barbers who go from house to house to dress the woman's hair in fantastic shape are carriers of syphilitic contagion. The pilgrims, usually sick persons, mostly syphilitic, known as *jun-rei,* to be met on the roads leading to holy places at all seasons of the year, clad in their white garments and in sandals, try to earn dispensation by visiting the sixty-six shrines of Kwannon, the Buddhist Goddess of Mercy. They are a dissolute set ofttimes, and carry from place to place where they stop the germs of their disease.

The public tea houses and archery galleries, run by girls who are in reality unlicensed prostitutes, transmit freely their syphilis and other venereal complaints. They are supposed to dispense only tea and *sake* as beverages to their customers, but they freely use their hands on males in a way not permitted in good society. The back rooms curtained off from the front ones are retiring places, hidden from the street's passers-by, where all sorts of orgies go on.

The peripatetic restaurateur carries the poison of syphilis on his saucers and cups.

Prolonged lactation of the mothers of Japan, three or four children of different ages nursing the same mother at the same time, because of the absence of cow's milk or other artificial baby food, and the habit of promiscuous wet-nursing are frequent methods of transmission of syphilis.

Many superstitions of Japan relate to women and the sexual act, or to the lives of prostitutes—male and female.

All the poetry of Japan is about courtesans, their dirty lives and suicide. All women, married concubines or prostitutes, carry razors in their toilet furniture as our negroes do as weapons, and women of Japan commit *hara-kiri,* not like men by cutting open their bellies, but, instead, cut their throats with the razor.

Spirit rapping in Japan is a prostitute profession and practiced only by women. Their "stock in trade" is a small box supposed to contain some mystery known only to the craft, of something less than a foot square. It is said that in the south a dog is buried alive, the head only being left above ground and food is then put almost within its reach, exposing it thus to the cruel fate of Tantalus. When in the greatest agony and near death, the head is chopped off and put in the box. Only the craft know what the box really contains. The medium has a small bow which she twangs incessantly on the box, and a small cup of water placed in front of her which is splashed toward the inquirer. If the person to be interviewed is living, a small piece of stick is used, if dead a leaf from a graveyard offering, called *shikimi,* is used to splash the water.

A prayer, an incantation and the spirit begins to speak in the box.

These boxes in miniature three inches long, made of wood, are carried by many women, but on sliding the lid—what do we behold—the genital organs of a man with the penis suspended and moving up and down.

This is the *spirit* which talks to the Japanese women.

A celebrated writing master of the century went on a holiday trip to *Nik-Ko.* He remained over night at a house occupied by one *Zenki.* Now, this *Zenki* was, as all his ancestors had been, custodian of the *Nikko Tengu,* "winged being." The traveler saw an immense boiler of rice being emptied hot into an enormous tub and taken to a room separate from the other apartments. Being curious he asked why this was done. Exercise some patience, said his host. Before long, strange noises were heard. Munchings, crunchings and smacking of lips, and then a scurrying about, followed by dead silence. The host then took him to the room where the tub was, and not a grain of rice remained. The tub was scraped clean, and with a solemn face and a warning sign in a tone of awe the host whispered: "Temmango has been to eat."

Tengu (Temmango) is the demon who does not molest good people, but those who worship him.

Scoffers and scoundrels, beware! He guards certain sacred places from sacrilege, especially mountains and shrines consecrated to Konpira (god of fire), the remedy (red hot iron) for chancres. He is invisible but is represented in offerings to temples by a rude countenance (that of the wine-bibber) and an enormous nose (prostitutes and concubines make it look like a penis) and he is accompanied by another mask, black, with an enormous beak; this latter is called *Karasu* (raven) *Tengu.*

The *Dai-Tengu* represents the male principle of the nation; the second or *Sho-Tengu* the female principle. These principles are carried about by women in the shape of *male and female* organs of generation as buttons or in boxes. The whole mind of the Japanese revolves around the sexual relations.

You could no more restrain the Japanese sexual appetite by Western ideas of morality than you could keep the sexes apart altogether. It is for this reason that ten per cent. of the Japanese army, as I am reliably informed, were really prostitutes, regularly enlisted and uniformed as soldiers.

The mortality table of venereal diseases alone of the Japanese army, if the truth is ever told about the sick lists of the Japanese *Samourai* of the Manchurian armies in the war with Russia, would of itself fill a large and interesting book.

There are altogether too many truth-despising, glory-chasing travelers of the medical and press-agent kind who, when they come back to civilized life in America, like to fill a column or two of newspaper space with some of their own Japanese-made inventions as to the wonders of Japanese sanitary and surgical science. They are no more reliable in fact than the stories told as to the absence of morbidity and mortality from the disease beri-beri.

Japanese statistics are totally unreliable—they are made to order to suit the object of the historical hour.—*American Journal of Dermatology.*

### THE CANCER PROBLEM.

We can not correctly speak of a renaissance of interest in the problem of cancer, because the mystery of malignant growths has continuously occupied the attention of the pathologist and clinician since the birth of modern medicine. It is true, however, that the past few years has witnessed more concentrated work on the problem of cancer than ever before, due probably, in large measure, to a general recognition that cancer is on the increase.

It is significant that the Oration on Surgery, by Dr. Joseph D. Bryant, at the latest A. M. A. meeting, in Boston, was devoted entirely to a consideration of the nature and progress of malignant disease. Only certain aspects of the problem were handled, but these were interestingly presented.

Regarding the increased occurrence of cancer, Bryant would attribute this to increasing longevity of life, resulting from sanitary advances, which brings many victims within the well-recognized devolution sphere of cancer.

The most interesting feature of the address was the consideration of relative occurrence of accessible and inaccessible malignant disease in males and females, the study being based on the statistics of the Imperial Cancer Research Fund. These disclose a most striking preponderance of inaccessible cancer at all periods of life, especially above twenty-five years—a fact which, Bryant says, startlingly suggests that, directly or indirectly, "subtler influences than external traumatism or infecting agents can exercise are active in the development and spread of cancer."

Again, cancer of the alimentary tract in the male is seven times commoner than in the female, a fact which, as Bryant points out, is inexplicable by any supposition of differing parasitic infection of alimentary substances in the sexes. Eighty per cent. of cancer in males affects the alimentary tract; in women, the same ratio affects the reproductive apparatus, including the breasts. These observations strengthen the general acceptance of traumatism in involuting tissue as a prominent etiologic factor. The remarkable preponderance of internal cancer—*i. e.*, in sites removed from gross external mechanical violence—makes it clear that traumatism, in this connection, is more elastic in its operation than hitherto considered, and includes, in the case of alimentary cancer, such factors as dietary abuses.

Touching the surgical treatment of cancer, Bryant pleads for early diagnosis and operation, emphasizing the occurrence of deep internal cancer with two and one-half times more frequency than accessible disease, entailing insuperable surgical obstacles in cases with extensive involvement.

Dr. Bryant's address could have profitably been extended to include many other phases of the cancer problem, such as a detailed review of the parasitic pathogenesis, and even more interesting and practical, the feature of spontaneous cure.

At the October, 1905, meeting of the New York State Medical Association, Dr. Harvey Gaylord, of Buffalo, whose work on the parasitic origin of cancer is remembered, read a paper on the "Spontaneous Cure in Cancer and Its Significance in Relation to the Ultimate Solution of the Problem of the Cure of Cancer," in which he referred to seventy unquestionable cures in fifteen hundred inoculated mice. None of these recovered mice could afterward be successfully inoculated with cancer; they became absolutely immune. It had been shown that there was a period in these cancers in mice in which the tumor can be made to retrograde with ease. If there was much hemorrhage and this blood got between the epithelial cells of the tumor, there followed an atrophy of the cells. It seemed that contact with the blood in the early stages took away the power of these cells of proliferation. Squeezing the tumor and causing hemorrhage into it produced the same effect. Again it was interesting that if the tumor had attained a certain size, the same procedure would cause it to grow rapidly. The injection, too, of adrenalin into the tumor caused it to grow rapidly. The histological portion of the tumor retrograding had a similar appearance as one retrograding under the action of the X-ray. In one experiment, one-half the mice were bled, causing the tumor to develop rapidly; the other half were not bled, and the tumor retrograded. The determining factor was whether the tumor did well after being interfered with, and this was a question of how much residual immunity there was. A great field for experimentation was now open in determining to what extent hemorrhages influenced the tumors. Loss of blood at operation might be advantageous, while prolonged anesthesia might be disadvantageous. The highest percentage of spontaneous cures occurred during the earlier stages of the growth. A good many mice had a natural immunity, and they could not be successfully inoculated.

A number of undoubted spontaneous recoveries had occurred in the human being, which he referred to, especially mentioning the case of Senger. This was a case of epithelioma of the cheek, opposite a carious tooth. Operation was recommended. There was much swelling in the submaxillary region and of the submental glands. The tooth was withdrawn, and in three months the tumor had entirely disappeared. Another patient presented himself with a similar condition of affairs. He operated upon a tumor the size of a cherry. There was the same submaxillary enlargement. The tooth was withdrawn. The sections of the epithelioma proved its nature without doubt. In the course of a few weeks the patient returned and the rest of the tumor had entirely disappeared. In going through the literature the best cases were found in the most malignant forms of cancer, the choroidal; there were eight cases of undoubted spontaneous cures of this malignant type.

At the same meeting, Dr. Samuel Lloyd, of New York City, urged an appreciation of the transmissibility of cancer. He considered cancer, in its first stage, a purely local disease, readily amenable to treatment as in cases of small epitheliomata of the face and lips. Later, however, it became constitutional and correspondingly difficult or hopeless to relieve.

Regarding transmissibility, he instanced the increasing ratio of cancer to the population, as shown by the health statistics of different countries. It was also shown that cancer was more common in thickly populated districts. In the poorer sections of Paris there were from 137 to 145 deaths per 100,000, while in the richer and cleaner sections there were but 75 deaths per 100,000. The same thing had been found to be true in other cities.

This was also true of country regions. He had had several experiences where the disease had no doubt been communicated from one individual to another. One man, an engineer, had cancer of the tongue, from which he died, and about a year later the night engineer, working in the same place, presented himself with cancer of the anterior pillar of the fauces. They had used the same drinking cup for several years. A man died of cancer of the stomach, and within two years Lloyd operated upon the wife for cancer of the breast; he had also operated upon a case of cancer of the stomach in a man whose brother had died of cancer a few months previously. A woman died of cancer of the breast, and two years later he operated upon her sister, who had nursed her, for cancer of the stomach. There was no previous cancer in the family. A physician's wife was operated upon for cancer of the stomach, the physician himself developed cancer in the lower jaw. Autoinfection with cancer was now a well-established fact. Gueillot believed that the epithelial origin of cancer would soon be admitted. Orth said, "Every cancer must be designated as an epithelioma." "The epithelial cells form the essential element of the cancer—they are not only the most important, but, indeed, the only important element." It was well known that we are constantly shedding our epithelial cells. If the normal were shed, so were the abnormal, and they might become a veritable cancer graft. In order to control the spread of cancer it became essential to prevent the dissemination of whatever element communicated the disease. The public should be educated to consider it contagious. Dressings removed from cancer cases should be immediately incinerated. The patient should be advised to avoid direct contact with others, kissing, etc. When a discharging wound was present, it should be covered with impervious dressing. In the second place, it should be remembered that cancer was engrafted upon points of ulceration, chronic irritation or developed in regions where abnormal epithelial tissue already existed. All contact with cancerous ulcers, and the common use of beds, utensils, drinking vessels, linen, etc., with cancerous patients should be avoided. All inflammatory lesions should be treated and cured, and all abnormal collections of epithelial cells should be removed.

It is evident that the last word has not been said on the infectiousness of cancer, and all this implies regarding immunity and measures other than surgical. Meanwhile, however, the general practician should heed the surgeon's plea for early diagnosis. This plea has already induced greater alertness and better results, evidences of which may be found in the interesting statistics given by Dr. Gerry Holden, in his excellent article in *The Georgia Practician* for April, 1906.—*Georgia Practician.*

---

### THE CAUSE AND TREATMENT OF SUMMER DIARRHEAS IN INFANTS.

BY J. P. CROZIER GRIFFITH, M.D.,
Professor of Diseases of Children in the University of Pennsylvania.

Recent years have turned the attention of physicians so strongly to the etiological influence of bacteria in many diseases that we are prone to forget that, even in those admittedly of bacterial origin, there may be other causes which, if not primary, are at least powerful in their action. This statement is certainly, I believe, true of diarrheal disturbances. The influence of the emotions in causing diarrhea in adults is well known. Severe diarrhea, or even dysenteric, conditions can be produced by a number of drugs, such as arsenic, corrosive sublimate, and different vegetable and mineral purgatives, and even many foods possess a powerful laxative action. Indeed, even when bacteria are known to be the cause in a given case, we must remember that it is after all the chemical action of resultant toxic substances produced, rather than by the mere presence alone of the bacteria, that the diarrhea is occasioned.

Confining ourselves more directly to the subject under discussion, the summer diarrheas of infancy, we must, I think, admit that we have at least two powerful etiological factors to consider: (1) bacterial growth, and the poisoning by toxins produced; (2) the action of hot weather upon the infant organism.

1. Bacteria as an etiological factor are generally recognized as the primary cause of these diarrheas. Milk is a peculiarly susceptible culture medium, and germs multiply in it in hot weather with enormous rapidity. Even in winter the average dairy milk is loaded with micro-organisms, and in summer it is only by the greatest care that their growth can be restrained in the best of milk within limits which an infant can safely manage. That it is particularly in summertime that the excessive infantile mortality from diarrhea develops, and that it is especially in bottle-fed infants that this occurs, proves the powerful influence which bacterial growth possesses. An additional proof is the great lessening of mortality among the poor to whom specially pure and properly modified milk is supplied.

It is to be remembered in this connection that not all bacteria in the milk are equally harmful to the infant. The ordinary germs of the lactic acid group, producing the souring of milk, are by no means so harmful as are certain others. This is well shown by the undoubted value of properly prepared buttermilk in diarrhea, a subject to which the physicians of the continent of Europe have given so much attention of recent years. In the preparation of this either a pure culture of lactic acid bacteria or a small portion of soured milk containing these germs is added to the milk before churning takes place.

The lactic acid bacteria clearly exert an inhibiting influence upon the growth of the germs of the bacillus subtilis group, of which the colon bacillus is one. It is this group which is particularly dangerous to infants; the more so, too, because the bacteria may abound in milk without producing any souring or other easily recognizable evidence of decomposition.

2. Yet there is certainly an action apart from the growth of germs which can be attributed only to the influence of heat upon the organism. This is shown, for instance, by the fact that even breast-fed babies are much more disposed to diarrhea in summer than they are in winter, and also by the well-known greater difficulty in checking summer diarrhea in bottle-fed babies in summer, even after all milk has been withdrawn from the diet. The sudden reappearance of severe diarrhea in convalescent infants not yet returned to a milk diet, corresponding with a sudden elevation of the temperature of the air, is another proof of the powerful action of heat upon the organism; as is the occurrence of cases of true cholera infantum, which seem to develop almost exclusively in the hottest weather. These conditions have been attributed to the greater increase of the bacteria within the intestine at such times. But there is no greater temperature present within the body in summer than in wintertime, and any increase in growth of bacteria in the intestines in the former season can be explained only on the ground that certain other influences permit of this. These influences are to be sought in part in the interference with the digestive functions, the result of the depressing effect of heat, which permits of this growth. In a certain number of cases, however, particularly those of sudden increase of diarrhea with extreme and rapid exhaustion of the infant contemporaneous with the existence of an excessively heated term, I am convinced, in spite of the general view to the contrary, that the cause is rather to be sought in the direct effect of heat upon the nervous, especially upon the vasomotor, system of the child.

The conclusion in brief is, then, that the very great majority of cases of summer diarrheas owe their origin to bacterial influence, but that the direct effect of hot weather upon the organism is generally a powerful secondary cause, and in some instances the chief one.

*Treatment.*—With the understanding of the underlying etiological principles the nature of rational treatment becomes apparent.

1. Most important is *preventive treatment.* First of all, milk should be as pure and as free from germs as possible. No amount of sterilizing of bad milk will make it good, since the poisonous chemical substances produced by the bacteria remain unchanged by heat. Next, to prevent further growth of germs and their entrance into the digestive tract, with further development there of toxins, a modified sterilization should be employed for all milk in summertime, with the exception of that produced by special dairies whose product is notably free from bacterial contamination. Even this milk is often sterilized in the hottest weather. Pasteurization is the method to be recommended—i. e., partial sterilization at temperature of 155° to 160° F. This process, however, requires exactitude. No rough and ready method answers. Milk cooler than the figures given is not benefited, and that heated above it has its natural ferments destroyed. Either a special pasteurizer, such as Freeman's, is required, or the employment of a thermometer throughout the process. One must remember, too, that while pasteurization destroys the bacteria, the *spores* of the subtils group are unaffected. Unless, then, the milk is carefully preserved *ice-cold* these spores, unchecked by the presence of lactic acid germs—since these have been killed—will multiply even faster than in raw milk. On this account commercial pasteurized milk, as supplied by dealers, is in my opinion absolutely to be shunned. No one knows how thoroughly the process has been performed, or how carefully the milk has been guarded afterward. The name gives to the users a false sense of security. All pasteurization should be done at home.

2. *Treatment of the attack* is simple in its principle, difficult in its application to different cases. The first indication is the absolute withdrawal of milk from the diet. This withdrawal should last one or several days, according to the case, such foods as cereal decoctions, albumen water, beef juice, and the like taking its place. If there is any reason to suspect that decomposed milk is still present in the digestive tract purgatives should be employed, such as calomel, castor oil, or magnesia in some form. Castor oil is one of the most useful, provided there is no vomiting and the stomach tolerates it well. It may be repeated on each of several consecutive days if required.

Then follows a return to the milk diet. The important principles governing this are, first, that it should not be hastily commenced; secondly, that it should be gradually accomplished. Where there is inflammation of the bowel, as indicated by continuance of fever after the evacuation and regulation of diet, and by straining and mucous stools, local treatment is most important. My preference is for large enemata of starch water, two level teaspoonfuls to the pint, given tepid. A quart or more should be employed, distending the colon as much as possible. Normal saline solution is good in many cases. Injections may be given once or twice a day. They should always be intermitted after a few days in order to see what nature will do without them. Sometimes they are not well borne. In some cases suspensions of bismuth in mucilage of acacia are of service, especially if the disease tends to become subacute.

I make little local use of the stronger astringents, such as nitrate of silver and tannic acid. Most children do not need them, and in any case they are only suitable for patients who have developed the chronic form of enterocolitis.

With regard to medication by the mouth, the first indication, as stated, is to empty the digestive tract. After this the employment of astringents is useful, such as bismuth subnitrate in doses of 6 to 10 grains every two or three hours, to which may well be added in many cases some vegetable astringent, such as kino or krameria, or the newer tannic acid compounds, such as tannigen and tannalbin, etc. Occasionally salol in small doses is of service, combined with the bismuth. The subgallate of bismuth may also be tried, as may beta-naphthol bismuth, but I have not found them generally superior to the older preparation.

Where fever has ceased and watery diarrhea persists opium is invaluable. We have now reached a stage where the irritation caused by food has passed and we are dealing with a too rapid intestinal peristalsis as a prominent factor. Many an infant will fail to respond to treatment unless opium is used. Caution is always necessary, both in the selection of the proper time and the grading of the dose. Many diarrheal cases in greatly debilitated states are peculiarly susceptible to opium, and the initial dose should always be small. In the choleraic cases opium is absolutely necessary to check the extremely rapid loss of liquid from the system. Here, too, enteroclysis is of value in order to add to the amount of liquid in the blood.

All this is the direct treatment of the disease. We have still to bear in mind such medication as general and cardiac stimulation, and the like. This is, however, purely symptomatic and need receive no special attention here, except to say that both alcohol and digitalis are generally well borne by infants in relatively large doses, and that enough must be used to obtain results. Here, too, must be emphasized the fact that many of the liquid peptonized food preparations on the market are strongly alcoholic, and that this must always be taken into account when giving them. Their chief action is that of stimulation, and their food value is very slight.

Finally we must consider the relief of the condition as far as it depends upon the high temperature of the weather. Nothing is more surprising than the rapid benefit which often follows removal of the child to a cooler climate. Untold good has been accomplished by seaside hospitals, river-steamboat charities, and the like. It is sometimes absolutely imperative to make the patient cooler. Among the poor in cities much can be accomplished by guarding from the heat of the midday sun, the passing the day in the parks, allowing the child to spend the evening in its coach in the open street, and the like. The use of water is important in this connection. Tub baths of a temperature of 90° or less, according to the case, may be given several times a day. In actual heat-stroke with great hyperpyrexia of the body much cooler baths may be employed. I have known of infants recovering through cold baths when the body temperature had reached even 110° F.

In all our treatment the child must be considered as an individual. No absolute rule can be laid down. The resourceful physician will often meet with success even under the most unfavorable conditions.—*Therapeutic Gazette, July* 19, '06.

---

FURUNCULOSIS vulvæ, even when it persists in spite of all other treatment, will usually yield to daily *scrubbing* with green soap and the application of a dressing of sublimate solution.—*American Journal of Surgery.*

### FRACTURE OF NECK OF FEMUR.

BY DE FOREST WILLARD, M.D.,
Surgeon in the Presbyterian Hospital, Philadelphia.

The treatment of fractures of the neck of the femur must necessarily vary with the character of the fracture and the age and condition of the patient.

*Impacted Fractures.*—Impacted fractures are not uncommon, and since the introduction of the X-ray the surgeon should be satisfied with a few diagnostic indications. Slight shortening with moderate eversion, diminished arc rotation, and relaxation of fascia lata are sufficient. After the X-ray shadow has been secured, the sooner such a fracture is dressed with plaster of paris casing, extending from the thorax to the knee (or in restless cases to the leg), the more rapidly and certainly will union take place.

In fractures of the neck with mobility, complete eversion, moderate shortening, lessened arc rotation, and relaxed fascia lata will be sufficient for diagnosis even though a skiagraph can not be secured. In any case of doubt an anesthetic will greatly assist in diagnosis. Treatment must vary in accordance with the age and condition of the patient. As a general rule (though subject to slight exceptions) union need not be expected in an individual over sixty-five; under that age measures to secure union should always be instituted with expectation of good result. With the patient under sixty, in good health, and where the skiagraph shows that the outer fragment can be brought down into good line with the caput, a steel nail or screw driven through the trochanter into the head will assist markedly in maintaining accurate apposition. The latter is best, as it can be buried and primary wound union obtained. The screw can be readily removed also after union has been secured and its presence is no longer needed.

In old and debilitated patients I make no attempt to secure union, simply making them comfortable, placing them upon a gas-pipe frame or tray with canvas support for trunk and legs, leaving a vacancy from the sacrum to below the anus. This allows them to be raised from the bed without disturbance, and the ends of the stretcher being supported, they can be kept clean and bed-sores avoided. Extension by pulley and weight and large sand-bags may be used intermittently, depending upon the amount of comfort derived from their employment, but they are not essential. The patient should be allowed to sit up as soon as possible, the chief attention being given to the condition of health.

The intermediate cases—those that bear confinement well—can be treated by a moderate amount of extension with pulley and weight, gas-pipe frame already alluded to being of great benefit in securing comfort and cleanliness. When confinement is not well borne, a plaster of paris from thorax to knee is of great benefit and allows the patient out of bed at an early date. This dressing is also of great service in mania a potu and delirious cases.

In many cases early release from bed may also be secured by adapting to the patient a Thomas posterior bar hip-splint with one hand around the thorax, another at the pelvis, two at the thigh, and one about the leg. With crutches and a high shoe, locomotion is readily secured; its use should be continued for many months. I have never tested the Ruth lateral double traction method, but it seems practical.

In cases that present themselves a year after the injury the problem is a difficult one. If the X-ray shows marked absorption of the neck, the best treatment will be to permit the patient slowly to form a false joint by voluntary movements. If, however, the head, trochanter, and neck are in fair line with the joint, the fragments may be screwed or nailed together. An open operation with fixation of the fragments requires so much manipulation that it is a serious matter and is not advisable, provided the patient has already secured a fair amount of fixation and is able to move about without serious pain.

In cases in which it is decided to secure a false joint, an apparatus with leather encasement of pelvis and body, with another stiff leather band encircling the thigh, will be very helpful. These broad bands may be connected by a joint which can be unlocked for the sitting posture and locked when the patient is erect.—*Therapeutic Gazette, May,* 1906.

### SUPRARENAL GLANDS AND THEIR RELATION TO CLINICAL MEDICINE.

Theodore G. Davis reviews the recent literature and thinks that adrinalin has two therapeutic indications—as a local hemostatis and to raise blood-pressure.

He concludes that the suprarenal gland is essential to life. It furnishes a product which, entering the blood, acts, directly upon the cells, increasing their irritability and contractility. This action is most manifest upon the muscle cells of the heart and peripheral vessels. It raises arterial tension to a high degree by increasing the force of the heart, by increasing the peripheral resistance, and by lessening the elasticity of the arterial walls; three factors pointed out by Janeway (53) as essential to the production of vascular hypertension; the fourth, volume of blood in circulation being influenced by the fluids ingested.

If there be continued excess of suprarenal secretion changes occur in the vessel walls corresponding to the pathological condition known as arteriocapillary fibrosis and atheroma, even to the extent of calcification, and I believe we are warranted in concluding that hyperepinephria is causative factor in their production. And, lastly, I desire to call your attention to an unrecognized etiological factor in the production of disease of the vascular system, namely—the sexual or procreative function. My attention was directed to this while studying the embryology and histology of the suprarenal glands. Its embryologic source (from the Wolfian bodies), the arrangement of muscle fibres in the walls of the veins, and the sinusoids, are suggestive of an erectile tissue, and close relation to the generative apparatus, and I know of no physiological function during which vascular tension is greater than during the sexual act. Excessive venery, then, must be looked upon as an important causative factor. From this viewpoint, syphilis is a concomitant disease.

Alcoholism and other intoxications are associated diseases; for the use of intoxicants, in some form, for the purpose of modifying vascular tension, has been common to all people from the earliest ages.

That complexus of symptoms known as neurasthenia has a physical basis in cell exhaustion. In the earlier stages it is accompanied by vascular hypertension, and Federn, of Vienna, states "all its manifestations disappear when the hypertension is controlled."

I would like to consider with you the rational treatment of vascular hypertension by rest, exercise, massage over the cardiac region, warm or hot baths, saline purgatives, especially sodium sulphate. Alkalies, which lessen the activity of the suprarenal secretion, of the thyroid, which opposes its action, and to give you a physiological reason for the good effects obtained by the use of iodides. I should like to point out the abuse of the nitrites, the hypnotics and narcotic drugs, but time does not permit.—*New York Med. Jour., August* 11, 1906.

---

### SKIN FOODS AND MASSAGE CREAMS.

These, when ideal, should be totally absorbed by the skin, no trace of grease or greasy feeling remaining after the massaging process. This can only be attained by the omission of grease altogether or thoroughly saponifying the oily ingredients. The most satisfactory preparation would be one of the massage creams containing casein as a base. The following is a formula for improved massage cream: Glycerine 1 ounce, borax 2 drachms, boracic acid 1 drachm, oil rose geranium 30 drops, oil anise 15 drops, oil bitter almond 15 drops, milk one gallon. Heat the milk till it curdles and allow it to stand twelve hours. Strain it through a cheese cloth and allow it to stand again for twelve hours. Mix in the salts and the glycerine, and triturate in a mortar, finally adding the odors and the coloring. The curdled milk must be as free from water as possible in order to avoid separation. Sometimes milk will not curdle as readily as at other times, but the addition of one ounce of water of ammonia to the gallon will hasten it. Take a gallon of good milk, add one ounce of ammonia water, heat (not boil), allow to stand twenty-four hours, and no trouble will be experienced in forming a good base for the cream.

If the lanolin and oil preparations are preferred the following formula, the favorite of Sands, the eminent dermatologist, should be tried. This is a special massage base or skin food: Snow-white cold cream 4 ounces, lanolin 4 ounces, ol. theobroma 4 ounces, white petrolatum oil 4 ounces, distilled water 4 ounces. In hot weather add spermaceti 1½ drachms, white wax 2½ drachms. In winter the two latter are left out and the proportion of cocoa butter is modified. Prepare and perfume in proportion same as cold cream.

Another good formula for facial massage cream: Warm milk to about 40° C. Add a small amount of ammonia water; allow the mixture to stand for twenty-four hours, when the fat which has risen to the top should be skimmed off. To the remaining liquid add acetic acid to excess to precipitate the casein. Collect the precipitate on a straining-cloth or filter. Wash the precipitate well with water till the washings are no longer acid. Allow to drain thoroughly. While precipitate is still slightly moist, add a small quantity of glycerine and rub the mixture in a mortar until a smooth paste is obtained. Add about two drachms of boracic acid to each pound of cream. Incorporate thoroughly about two drops of bitter almond oil or other flavoring agent, and carmine to give a pink or flesh color to the cream. Keep it in well-stoppered bottles.

Following is a good skin cream: Lanolin 2 ounces, cottonseed oil (bleached) 3 ounces, castor oil 2 ounces, spermaceti one-half ounce, white wax one-half ounce, borax one-half drachm, aqua rosa 3 ounces. Perfume and color.

Good wrinkle cream: Oil amygdalæ dulce 3 ounces, tr. benzoin 2 drops, oil cocoa 1 ounce, lanolin 1 ounce.

Another skin food: Oil amygdal. dulce 2 ounces, lanolin 1 ounce, ext. hamamelis conct. 2 ounces, oil cacoa 2 ounces, mutton tallow 1 ounce, tr. benzoin 1 drachm, ext. rosa alb. one-half drachm. Beat oils to cream, then slowly add tr. benzoin drop by drop, then witch hazel and perfume.

Another good wrinkle cream: Lanolin 1 ounce, white wax 1 drachm, spermaceti 1 drachm, oil olive 1 ounce, camphor (gum) 1 ounce.

Following is what is called a pore food: White wax one-half ounce, spermaceti one-half ounce, almond oil (sweet) 1 ounce, vaseline one-half ounce. Melt and stir. If too thick, add almond oil.

---

### STASIS HYPEREMIA IN ACUTE SUPPURATIVE PROCESSES.

Since the early part of the year 1905, when Bier first recommended stasis hyperemia in the treatment of acute suppurative inflammations, Herhold (*Munchener medicinische Wochenschrift*, Feb. 6, 1906) has employed it in thirty-five cases, of which fourteen were felons, five suppurative tenosynovitis, fifteen furuncles and one a cellulitis of the thigh.

The boils were treated by covering them with a glass dome from which the air was exhausted. In the remaining cases circular constriction of the extremity was applied above the seat of the lesion. To prevent the formation of blebs and vesicles when employing circular constriction the skin should be protected from the rubber band by a few turns of a primary muslin bandage.

The amount of constriction must be kept under control for at least the first ten minutes. It should be sufficiently great to abolish the pain and yet not cause blueness and coldness of the extremity. If properly applied the skin is somewhat warmer and redder than normal, and there is no numbness of the digits. The amount of the constriction should be regulated again in the afternoon.

The felons were opened by a small incision as soon as a focus of pus manifested itself beneath the skin, and the congestion was continued. No drainage nor packing was used in the wound, but a moist dressing was applied to the surface. The patients stated that pain subsided as soon as the congestion was instituted. On the following day if pus had not already been manifest it was found in a small drop at the tip of the finger. This was evacuated, and within two or three days more the central slough was so softened that gentle pressure caused it to be exuded, whereupon there was left a simple granulating wound.

In treating boils mild suction was employed for fifteen or twenty minutes twice daily. Boils in which pus had not yet formed rapidly disappeared. In cases in which pus was present the overlying skin was first incised, and then suction during the following days aspirated pus and blood, and boils were cured without further treatment. With large furuncular abscesses an incision at least one centimeter long was necessary to secure the desired results. The painful packing of these wounds is rendered unnecessary by this method. The boil is protected simply by placing over it a small piece of perforated gauze which is held in place by two small strips of adhesive plaster.

In two of the fourteen cases of felon stasis congestion caused an increase of pain and inflammatory signs, so that this treatment was discontinued; in one the patient was cured without suppuration; and the remaining eleven pursued a very favorable course. The average duration of treatment in the twelve cases was eleven days.

In one of the fifteen cases of furunculosis, stasis treatment was discontinued in favor of local applications because of widespread outbreak of boils. The remaining fourteen cases were cured after an average of 9.2 days of treatment.

Of the five cases of suppurative tenosynovitis of the hand, treatment was ineffectual in one because of the great extent of the affection; in a second improvement during the first two days was followed by an increase in the severity of the symptoms; in a third case a preexisting extensive lymphangitis became worse at the site of constriction and the patient showed marked febrile reaction; in only two cases was treatment pursued till patients were cured, on the fourteenth and twenty-eighth days respectively.

The cellulitis of the thigh which developed secondary to a kick subsided within twenty-four hours after applying constriction, leaving only the lacerated wound for further treatment.

In conclusion, the writer states that stasis hyperemia in acute suppurative processes, particularly in furuncles and felons, gives very good results and should be regarded as an additional means of surgical treatment, but it is not a cure-all, and should be used only with caution and intelligent supervision in severe phlegmonous inflammations.—*Ther. Gazette.*

---

### CATAPLASMS.

Poultices should always be made by women, and should be hot, big, thick. If the necessary thickness makes it so heavy as to be painful to the patient, it can be made thinner and covered with cotton or oiled silk to retain the heat. It must be replenished often to keep it hot and not allowed to get cold and sticky. The prime object is *moist* heat. The sufferings of the patient are often diverted from his real pain to the suffering induced from frequent application of a *hot* poultice. The following verse is expressive:

> Sweet faced nurse, 1ed hot poultice;
> Slaps it on and takes no notice.
> Patient yells and swears 'tis hot.
> Nurse just smiles and says 'tis not.

Cataplasma kaolinum is one of the additions to the new U. S. P. It is to take the place of the numerous clay preparations which are becoming household words among the laity, and should be prescribed in their stead by the profession. It consists of kaolin powder (fine) 577.00; boracic acid, fine powder, 45.00; thymol, 0.5; methyl salicylate, 2.0; ol. menth pip., 0.5; glycerine, 375.00; add 1,000. Heat kaolin in a suitable vessel 100° C. (212° F.) for one hour. Mix thoroughly with boracic acid, then glycerine, finally add thymol which has been dissolved in methyl salicylate and oil of peppermint and make homogeneous mass. Keep in air-tight container, which can be placed in hot water with the lid on to render it hot for application. Should be applied hot, covered with cotton and oiled silk, with bandage to retain, and allowed to remain twenty-four hours, when the putty-like mass will become dry and readily peel off. If an attempt is made to remove it before this time it will stick and be removed with difficulty. It should be applied one-half or one inch thick. It has a pleasant odor. The benefit is principally due to the heat and the hygroscopic action of the glycerine; the boracic acid, thymol and methyl salicylate render it mildly antiseptic. It is much neater than the ordinary poultice, is much less trouble to the nurse, gives the patient profane thoughts but once in twenty-four hours instead of ten or fifteen times, is quite efficient, and is a very valuable addition to the new U. S. P.

A poultice should be applied gradually and extend two inches beyond the affected surface. Should be discontinued as soon as the object is attained, as trouble may follow its too prolonged use.

The oiled silk jacket has in the pulmonary inflammations of children almost supplanted the time-honored flaxseed or onion poultice. It keeps the skin a uniform temperature, maintains a moderate degree of counter-irritation, and gives the patient a great deal of comfort. Poulticing in children is useful in local inflammations of the glands of the neck, the joints and in cellulitis situated in various parts of the body. In otitis harm often results from the too prolonged use of the poultice. Heavy poultices do harm in diseases of the chest of children by embarrassing the respiration. They are also harmful in this class if not administered by a skilled hand, keeping up a regular temperature and avoiding exposure in changing. Hot fomentations are more cleanly than poultices and much more easily changed. Wring a piece of spongio-piline or flannel out of water hot as can be borne and apply covering with cotton and oiled silk.

Charcoal poultice: Pulv. carbon, 16.00; micae panis, 60.00; aquæ bullientis, 300.00; pulv. linæ, q.s. Of benefit in gangrenous sores.

Alum poultice is made by adding 60 grains of powdered alum to the whites of two eggs, coagulating them. Coagulating and astringent.

To render poultices antiseptic, a very important thing, add 1 per cent. of phenol liquefactum to the water used in making the poultice.

Soap poultice: A paste of green soap spread on gauze is used in preparing surfaces for operation.

Yeast flour and water set to "rise," is a convenient and smooth poultice.

Powdered slippery elm and hot water poultice is peculiarly applicable to very sensitive surfaces. It is light, smooth and unctious.

---

### PHAGOCYTOSIS AND OPSONINS.

The writer states that at present we may accept as an established fact that phagocytosis of many bacterial and other cells by the leucocytes, in the first instance, is dependent on special substances, normal and immune, which become attached to the cells in question, and in some manner so change them that they are taken up readily by polynuclear leucocytes in vitro. Wright and Douglas have shown the presence in blood and other fluids of such certain substances, which they called opsonins. By means of experiments giving comparable results, it has been found that the variable factor is the serum, and not the leucocytes. The sera of the higher animals normally contain opsonin for many different bacteria. Thus, normal human serum contains opsonin for staphylococci, streptococci, pneumococci, meningococci, gonococci, influenza bacilli, diphtheria bacilli, anthrax bacilli, tubercle bacilli, typhoid and colon bacilli, the comma bacilli, the pest bacillus, and probably many other pathogenic and non-pathogenic bacteria. Whether this wide range of opsonic action is dependent wholly on a common opsonin, or on several more or less specific opsonins, has not been determined. It was later shown that the blood of certain animals immunized with blood contains a substance that, by acting on blood corpuscles, renders them subject to phagocytosis by leucocytes. Immune opsonins may be regarded as acting primarily on the bodies against which the animals have been immunized. Phagocytosis is essentially amœboid motion, enclosing, or partly enclosing, the object within the phagocyte.

The action of the opsonins may be defined to consist in so changing bacteria and other cells that these, by chemical, electrical, or mechanical means, diminish the surface-tension of leucocytes, and thus bring about phagocytosis. Again, there also takes place under certain circumstances the production of specific antiopsonins, which may diminish or inhibit normal phagocytosis. The important question is the value of the opsonic index in the successful treatment of infections by means of the inoculation of bacterial vaccines. These vaccines should be administered when the opsonic is high, and in properly adjusted and interspaced doses; in other words, by controlling the effects by means of the opsonic index, to maintain the antibacterial power of the blood at a high level. The reason why opsonic action of normal and immune serum is to be regarded for the present as connected with distinct bodies may be summarily stated as follows: 1. Normal serum may possess lytic power, but not opsonic, and vice versa. 2. Immunization may give rise to opsonic substances, but not to lytic or agglutinating. 3. Heat may destroy opsonic power, while the lytic amboceptors remain intact, and vice versa. Ludwig Hektoen (*Journal of the American Medical Association, May* 12, 1906).—*Monthly Cyclopedia of Practical Medicine, July,* 1906.

---

### MENSTRUATION AND PREGNANCY.

Bond, as a result of his experiments on rabbits, has arrived at the following conclusions regarding menstruation and pregnancy: The presence of functionally active ovarian tissue is necessary for the uterine function, or that portion of it which is concerned with the preparation by the endometrium of a suitable nidus for the imbedding of fertilized ova. The presence of the uterine or endometrium tissue is not, on the other hand, necessary for the carrying on of ovarian function, either ovulation or the production of the internal secretion associated with œstrus. One function of the endometrium in the anœstrous state is the secretion of a saline watery fluid of low specific gravity, containing a large amount of chloride of sodium in solution. There is some antagonism between this endometric function or saline secretion and that portion of the internal secretion of the ovary which is specially concerned in producing procestrous changes in the endometrium preparatory to the imbedding of fertilized ova. That portion of the internal secretion of the ova which is, in fact, associated with the growth of corpora lutea. The saline uterine secretion is associated with katabolic, the ovarian secretion with anabolic changes. The mechanism by means of which the ovary obtains its stimulus from the stimulated endometrium consequent on the occurrence of pregnancy is a circulatory and not a nervous mechanism. In all probability some substance is manufactured by endometrium or trophoblast which reaches the ovary by way of the blood stream, and is also concerned with the increased activity of the mammary glands. The bilateral ovaries may be regarded as one gland as far as the function of the endometrium is concerned. The whole procestrous process is of the nature of a preparation for the attachment of an embryo. In addition to ovulation the mammalian ovary elaborates an internal secretion which at recurring periods is the cause of procestrous and æstrous. The corpora lutea form a ductless gland which is necessary for the nutrition of the trophoblas during the early stages of pregnancy and subsequently atrophiles. The established facts as regards the uterus are: (a) That the endometrium has a secretion peculiar to the ancestrous state; (b) that some substance is elaborated by the pregnant uterus which stimulates the growth of corpora lutea in transplanted ovaries.—*British Medical Journal, July 21, 1906.*

---

### Milk as a Carrier of Infection.

In a general way there are four distinct periods when the milk may be polluted from the animal to the table. There is always, as a rule, pollution at the time of milking. This may be caused by unclean cows, upon whose flanks undoubtedly many of you have seen layers of filth and fecal matter; from foul air in a poorly, if at all ventilated barn; filthy udders; the dirty hands or dirty, dust-laden apparel of the milker.

The greatest risks in transit are from unclean utensils and milkcans. With such a condition great numbers of bacteria remain in cans from previous contents, and when fresh milk is introduced the bacteria in a short time begin to multiply.

The increase is very rapid, particularly so in summer if shipment is delayed and temperature increased, as temperature has much to do with the multiplication of bacteria.

There are many ways in which milk may be polluted in the milkshop, namely, from street dust when cans are left open, from the air, the shop, the dirty hands or dust-laden clothing of the one who may handle the milk for customers purchasing same, from impure ice which may gain entrance from improper cooling apparatus and methods of handling. Not the least of all, the addition of water to the milk previously sterilized will add to the bacteria already contained. This is a common means of pollution.

One of the most common sources of the pollution of milk is in the home. Here you will find all kinds of dust, particularly in the tenement, foul air and abundance of heat. If there is no ice-chest the milk may be allowed to stand in some sort of a receptacle, pan or pitcher to receive the bacteria and other organisms, plus dust, both organic and inorganic.

No matter how pure the milk may be when delivered to a home where such conditions exist, it is surprising to see the effect on children under two years, who depend upon milk for nourishment. Here the sanitarian can do nothing but educate the people in the proper way to care for milk.—*W. W. Brand, Ohio Sanitary Bulletin, in Journal Physical Therapy, May,* 1905.

---

### OBSTETRICS AND GYNECOLOGY.

BY E. S. MCKEE, M.D., CINCINNATI.

*An Unmentioned Means of Puerperal Infection.—Greene* (*N. Y. Med. Journal*), reports following unique case as to the course of puerperal infection:

The husband was handling hides for a hide merchant, but as his great ambition was to become a doctor, which ambition he hoped soon to realize, he was in the habit of making frequent examination per vaginal to note the progress of labor or the course of pregnancy with the aid of a book on obstetrics. Labor was perfectly normal, but two days later the patient was taken with a chill, which was followed by a terrible attack of puerperal fever. The woman eventually recovered, but was left totally blind in one eye and partially so in the other from an intercurrent attack of optic neuritis. Greene is convinced that the puerperal infection came from germs carried from the filthy hides which the husband was handling.

*Treatment of Prolapsed Ovaries.*—Gardner recommends an operation which consists in shortening the elongated ligament ovarii by a couple of fine silk stitches. The first stitch takes a light but firm hold in the uterus near the lower border of ovarian ligament; it is then continued through a portion of the ligament and is inserted firmly in the ligament near the ovary. The second stitch is placed the same way, but near the upper border of the ligament. When these stitches are tied the ovary is brought up near the uterus, but still retains a limited mobility independent of the uterus, and a complete mobility with the uterus. Retrodisplacement of the uterus, if present, should be operated on at the same time.

" 'Tis my delight
To be out at night,
For I'm an accoucheur."

---

### VARIATIONS IN THE FAT PERCENTAGE OF MOTHER'S MILK AS A FACTOR IN FEEDING—A FOREWORD.

L. Taylor Jones believes that the importance of mother's milk can not be overestimated. A physician should feel that he is taking the baby's life in his hands in lightly changing from breast milk, and should so impress the mother. Besides the immediate danger, which at times is not so great, it lessens the stamina for later years. A right start in anything is essential, but nothing is more important than a right start in life.

If there is some disturbance to the nursing infant, the breast milk should be examined, unless some cause, like tuberculosis, is at once recognized. It is not long since patients were pronounced anemic upon looking at them, but to-day the hemoglobin must be estimated. So must it be with the breast milk.

Fat is an important factor if only for its variability.

The importance of the fats has increased lately since the Breslau investigators gave them such an important rôle in infantile atrophy (marasmus).

For the most part fat gradually increase in amount from the beginning to the end of a feeding, with occasionally a dip down at the end. As yet there is no proof that their increase is arithmetical. A baby that needs more fat than it is getting can easily be put to the breast after some milk has been pumped out.

A fat percentage, within a few tenths of a per cent. of the average may be obtained by taking equal specimens from the beginning and end of the feeding and examining the mixture. This is entirely practicable clinically and should be done.—*Archives of Pediatrics, July,* 1906.

---

### SUCCESS: THE SURGICAL DESIDERATUM.

Dr. A. Ernest Gallant, of New York City, read this paper at the Boston meeting of the A. M. A., summarizing as follows the factors which, in appropriate cases, helped to bring about surgical success:

1. A most careful consideration of antenatal and postnatal diseases, and the chronicity or acuteness of the present condition before deciding to operate or not to operate.

2. The liberal use of water by mouth and rectum, before, during and after operation. The free application of tincture of green soap, rubbed into the skin until dry when preparing the field of operation and the hands of the operator and assistant.

3. Minimum morphine, before anesthesia; the ether administered by the patient herself, in the first stage; care that the head is properly elevated; and a minimum quantity of ether employed.

4. A reasonably short incision, a reasonably quick operation, and a reasonable number of sutures to close the wound.

5. After operation the careful prevention of chilling the body; the cautious use of morphine and sedatives; lavage to allay undue vomiting; and colonic massage and kneading for relief of distressing intestinal distention. Continuous rectal irrigation for tympany.

6. For the invigoration of the tired patient, and to prevent muscular atrophy and cardiac weakness, general massage daily, with passive and active movements while in bed and calisthenics when first about.

7. For the prevention of hernia and enteroptosis or support of prolapsed organs we apply the abdominal "stock," Rose's plaster bandage or a special corset, put on while the patient is in the dorsal-inclined posture.

---

### PREMENSTRUAL FEVER IN TUBERCULOUS WOMEN.

Kraus (*Wien. Med. Wochenschrift*) remarks a rise rarely exceeding a few tenths of a degree in corresponding to the menstrual cycle, in a number of perfectly healthy women, occurring at regular intervals, isochronous, but not synchronous, with the catemenial flow. It usually precedes the flow a day or two, rarely a week. In tubercular women, on the other hand, he found a distinct regular rise very common, appearing in at least two-thirds of the women in a sanitarium under his care. In healthy subjects the rise was usually noted a day or two before the show of blood, but it often happened a week before, and in some instances two weeks. In the latter cases the rise, of course, appeared about midway between the periods. In each of these tubercular subjects the rise in temperature, whether a long or short time before the menstrual flow, remained regular as to the time of its occurrence. It was only in very rare cases that there was a rise synchronous with the show. The rise was usually gradual, falling when the show appeared and attended with little constitutional disturbance. It nearly always reached its highest point early in the afternoon. His most important result from these observations was the discovery of the fact that the periodical rise of temperature bore no relation to the gravity of the general tuberculous infection, and indeed in some instances this periodical rise led to the detection of the tubercular condition.

---

### THE CONSERVATIVE SURGERY OF THE OVARIES.

Dr. Edward Reynolds, of Boston, read this paper before the A. M. A. at Boston. If cystic disease of one ovary existed it tended to appear in the other. If one ovary was affected it was conservative surgery to remove the affected one, leaving the other. If both were affected conservative surgery called for the removal of both, saving as much ovarian tissue as possible. Malignant neoplasms meant radical operation. After the child-bearing age both ovaries should be removed if affected, the disadvantages of castration in such case being of minor importance as compared with the probability of second operation. Normal ovarian tissue could generally be found in benign tumors, and effort to preserve as much as possible of this normal tissue should be made. Ovarian tumors could conveniently be classified as cysts originating from the corpora lutea, dermoid cysts, Graafian cysts, and enlarged sclerotic ovaries. Cysts of the corpora lutea would slip out through an incision, leaving practically normal ovarian tissue. Dermoid cysts and the larger cysts of the Graafian follicles must usually be excised. Enlarged sclerotic ovaries may be treated by puncture and removal of the lining membrane, with or without suture; resection; bisection of the ovary and multiple puncture or resection; and scarification or multiple incisions of the surface.

---

### DIFFUSE SEPTIC PERITONITIS.

In the *Annals of Surgery,* February, 1906, Dr. Robert G. LeConte gives the following resume of the treatment of diffuse septic peritonitis:

The rapid elimination of the cause of the peritonitis, whether it be a perforation of the bowel, a gangrenous appendix, a ruptured pus tube, etc. This must be done with the least possible handling of the peritoneal contents.

Drainage by tube of the lowest portion of the pelvis through a suprapubic opening, and free drainage through the operative incision.

The elimination of all time-consuming procedures at the time of operation.

The semi-sitting position of the patient after operation, so-called Fowler position.

The absorption of large quantities of water through the rectum, which reverses the current in the lymphatics of the peritoneum, making the surface of that membrane a secreting instead of an absorbing one, and also markedly increasing the secretion of urine.

The prevention of peristaltic movements of the intestines by withholding all food or liquids by mouth, and perhaps by the administration of opium.

---

### ALBUMIN IN THE URINE.

Tunis draws the following conclusions: From any point of view the term physiological albuminuria is misleading, unsatisfactory and inadequate. As long as albumin remains in the urine an individual is not in a normal condition. The mortality among such subjects is higher than among those who are free from such a condition. The actual mortality rate can be approximated by comparing the records of several large life insurance companies through a period of twenty years. The prompt means of discriminating between the transient forms of albuminuria and those of real clinical significance may be found by such a test as the calcium lactate. A faint trace of albumin in the urine of one who has passed middle life is often of greater significance than a decided trace by directing attention to the presence of casts which might otherwise have been overlooked. For practical purpose the heat and nitric acid test for albumin is the best one, and the careful use of Roberts's solution the most satisfactory control test in doubtful cases. For the proper diagnosis and prognosis too much stress can not be laid on the thorough consideration of the clinical condition, as a whole.—*Journal American Medical Sciences, July, 1906.*

